# Decolourisation of Synthetic Dyes by Endophytic Fungal Flora Isolated from Senduduk Plant (*Melastoma malabathricum*)

**DOI:** 10.5402/2013/260730

**Published:** 2013-07-22

**Authors:** Ngui Sing Ngieng, Azham Zulkharnain, Hairul Azman Roslan, Ahmad Husaini

**Affiliations:** Department of Molecular Biology, Faculty of Resource Science and Technology, Universiti Malaysia Sarawak, 94300 Kota Samarahan, Sarawak, Malaysia

## Abstract

A total of twenty endophytic fungi successfully isolated from *Melastoma malabathricum* (Senduduk) were examined for their ability to decolourise azo dyes: Congo red, Orange G, and Methyl red and an anthraquinone dye, Remazol Brilliant Blue R. Initial screening on the glucose minimal media agar plates amended with 200 mg L^−1^ of each respective dye showed that only isolate MS8 was able to decolourise all of the four dyes. The isolate decolourised completely both the RBBR and Orange G in the agar medium within 8 days. Further quantitative analysis of the dye decolourisation by isolate MS8 in aqueous minimal medium showed that isolate MS8 was able to decolourise all the tested dyes at varying levels. Dye decolourisation by the isolate MS8 was determined to be 97% for RBBR, 33% for Orange G, 48% for Congo red, and 56% for Methyl red, respectively, within a period of 16 days. Molecular identification of the fungal isolate MS8 using primer ITS1 and ITS4 showed that isolate MS8 shared 99% sequence similarity with *Marasmius cladophyllus*, a Basidiomycete. The ability to decolourise different types of dyes by isolate MS8 thus suggested a possible application of this fungus in the decolourisation of dyestuff effluents.

## 1. Introduction 

There are more than 100,000 colours of synthetic dyes produced commercially, and over 7 × 10^5^ tons of dyes are produced annually worldwide [1]. Dyes are used throughout the world in textile, paper, cosmetic, pharmaceutical, and food industries and also used as additives in petroleum products [2]. Synthetic dyes are extensively used particularly in the textile and dyeing industries. In the process of dyeing, about 15–20% of the dyes used for dyeing do not bind to the fibres and are lost in the effluent [2]. It has been estimated that about 280,000 tons of textile dyes are discharged in such industrial effluents every year worldwide [3].

The discharge of textile dyes into rivers or lakes is the most visible sign of water pollution because several dyes are visible even at a low concentration of less than 1 ppm [4]. In addition to changing the colour of water, the presence of dye in the receiving water bodies also impedes sunlight penetration that in turn decreases photosynthetic activity, dissolved oxygen concentration, and water quality and causes acute toxic effects on aquatic flora and fauna [5]. Many synthetic dyes are also toxic and carcinogenic to aquatic and human lives because they are made from compounds such as benzidine and other aromatic compounds [6]. Reductive cleavage of azo dyes, which comprises about 70% of all dyes used, also resulted in the production of amines that are mutagenic to human and are also retained in the anaerobic compartment of the lower intestine by intestinal microflora after ingestion of azo dyes [7–9]. Therefore, industrial effluents containing dyes must be treated prior to their discharge into the environment.

Among industrial effluents, wastewater from textile and dyestuff industries is one of the most difficult to be treated since the dyes used are usually synthetic and contain complex aromatic molecular structures making them more stable and difficult to degrade [10]. Conventional wastewater treatment plants using activated sludge treatment are unable to treat dye-containing wastewater. It has been estimated that up to 90% of reactive textile dyes still persist even after the treatment [11]. Several physical and chemical methods including membrane filtration, adsorption, ion exchange, ozonation, flocculation-coagulation, and oxidation have been used to treat dye containing wastewater. However, due to high cost involved, disposal problems, and less adaptable to a wide range of dye wastewaters, most of these methods have not been widely applied [6, 12]. Bioremediation of dye containing effluents using effective dye degrading microorganisms is still seen as an attractive alternative solution due to its low-cost, environmentally friendly, and publicly acceptable treatment technology [6].

 White rot fungi have been shown to be able to degrade a wide range of organic pollutants including synthetic dyes [13–16]. In recent years, many studies have focussed on the use of white rot fungi to decolourise synthetic dyes due to its ability to produce nonspecific, ligninolytic enzymes [16–18]. Dye decolourisation, in particular by the white rot fungus *Phanerochaete chrysosporium*, has been intensively studied, and the degradation pathway for sulfonated azo dye by this isolate has also been elucidated [6, 9]. However, there is no report on the use of endophytic fungi for the decolourisation of dye. Most studies on endophytic fungi have focussed on studying its relationship with its host and its diversity and also to isolate bioactive compounds of medical value from these fungi [19–21].

In this view, the present investigation is an attempt to find out and examine the possible application of very little studied endophytic fungi isolated from *Melastoma malabathricum* to decolourise several synthetic dyes belonging to two major dye groups widely applied in the dyeing industries. Here we report the screening and isolation of dye decolourising endophytes on agar medium and dye decolourisation analysis to select the best isolate that decolourises synthetic dyes.

## 2. Materials and Methods

### 2.1. Isolation of Endophytic Fungi

The endophytic fungi used in this study were isolated from the stem of a healthy flowering plant, *M. malabathricum*. Plant samples were collected from the surrounding areas of Universiti Malaysia Sarawak (UNIMAS) and Kota Samarahan campus and processed immediately after collection. The plant samples were washed under running tap water to remove debris and air-dried before being cut into 5 cm^2^ in diameters. In order to eliminate epiphytic microorganisms, the samples were surface-sterilized [21, 22] by immersion in 5% (v/v) Clorox for 5 minutes, followed by 70% (v/v) ethanol, and rinsed twice with sterilized distilled water. The surface sterilized samples were blot-dried using sterile filter paper and then aseptically cut into 2 cm^2^ in diameters. The pieces of stems were then transferred aseptically onto potato dextrose agar (PDA) (Merck, Germany) plates (3 pieces per Petri plate) and incubated at room temperature for a period of 2 weeks. The plates were observed daily, and hyphal tips of developing fungal colonies were subcultured individually. Fungal isolates were distinguished based on its colony morphology.

### 2.2. Dye Decolourisation Experiments

The isolated endophytes were grown onto glucose minimal (GM) agar plates and initially screened for their ability to decolourise anthraquinone dye (Remazol Brilliant Blue R, RBBR) and three azo dyes (Orange G, Congo red, and Methyl red). The GM agar medium contained (g/L): K_2_HPO_4_, 1; ZnSO_4_·7H_2_O, 0.01; CuSO_4_·5H_2_O, 0.05; MgSO_4_·7H_2_O, 0.5; FeSO_4_·7H_2_O, 0.01; KCl, 0.5; glucose, 10; NaNO_3_, 3; and agar, 20. The pH of the agar medium was adjusted to 5.5 before being autoclaved at 121°C for 15 minutes. Dyes were added into the agar from a stock solution to a final concentration of 200 mg L^−1^. The agar plates were inoculated with a 5 mm^2^ agar plug from a 7-day old fungal culture and incubated in the dark at room temperature. Uninoculated plates with the respective dyes were used as the control. Each isolate was prepared in duplicates. Plates were regularly monitored and observed for visual disappearance of colour for a period of 16 days.

The best dye decolourising isolate was selected for further dye decolourisation in liquid GM medium. Dyes were added to the 20 mL GM liquid medium in 100 mL Erlenmeyer flask to a final concentration of 200 mg L^−1^. Each flask was inoculated with 2 pieces of 5 mm^2^ agar plugs from a 7-day old fungal culture and incubated in the dark at room temperature under static condition. Flask with the respective dye and no fungal inoculum was used as control. Each culture condition was prepared in triplicate, incubated for a period of 16 days, and sampled at 4-day interval. During the sampling, each culture was harvested and centrifuged at 6000 rpm for 10 minutes to separate the fungal mycelium from the culture medium. Fungal biomass was determined by drying the fungal mycelium to a constant weight at 70°C. Dye decolourisation by the isolated fungus was measured by monitoring the absorbance of each dye in the culture medium at its respective maximum absorption wavelength (595 nm for RBBR, 475 nm for Orange G, 497 nm for Congo red, and 520 nm for Methyl red) using a UV-Vis spectrophotometer (Libra S12, Biochrom). 

Percentage of decolourisation was calculated according to the following formula:

(1) $$\begin{array}{c} \text{percentage of decolourisation }\left(\%\right)=\frac{A_{c}-A_{s}}{A_{c}}\times 100, \end{array}$$

where *A*_*c*_ is the absorbance at the maximum absorption wavelength of dye in the control flask at time, *t*, and *A*_*s*_ is the absorbance at the maximum absorption wavelength of dye in the sample flask at time, *t* [23].

### 2.3. Fungal Characterisation and Identification

The selected fungal isolate was identified by morphological characteristic as well as comparison of internal transcribed spacer (ITS) sequences. The morphological appearances of the selected fungal isolate were characterized based on mycelium colours, growth patterns, and structure of fruiting bodies. Genomic DNA of the selected fungus was extracted according to the method of Cubero et al. [24]. Extracted fungal DNA was then PCR-amplified using universal primer pair of ITS1 and ITS4 under the following condition: initial denaturation at 95°C for 5 minutes, 30 cycles of denaturation at 95°C for 1 minute, annealing at 55°C for 1 min, extension at 72°C for 1 min, and a final extension at 72°C for 7 minutes. PCR products were then purified and sequenced. Closely related sequences of the isolates were retrieved from the NCBI GenBank database. A neighbour-joining tree [25] was constructed, and the distances between sequences were calculated [26]. Bootstrap analysis was performed with 1000 replications to assess the confidence limits of the branches [27].

## 3. Results and Discussion

### 3.1. Isolation of Endophytic Fungi

A total number of twenty endophytic fungi were successfully isolated from the stem of *M. malabathricum*. These fungi were named according to the source from which they were isolated and followed by a number (MS1–20 with M = *Melastoma* and S = Senduduk).

### 3.2. Dye Decolourisation on Agar and Liquid Medium

Dye decolourisation activity of the isolated endophytic fungi was screened using an agar plate method with decolourisation observed by the production of clear halo. All of the tested fungi were able to grow on the dye containing minimal agar medium, and 14 of the fungal isolates were able to decolourise at least one of the dyes (Table 1). Among the 20 tested fungi, 13 isolates were able to decolourise RBBR, 2 isolates decolourised Orange G, 13 isolates decolourised Congo red, and 9 isolates decolourised Methyl red. 

**Figure 1:**
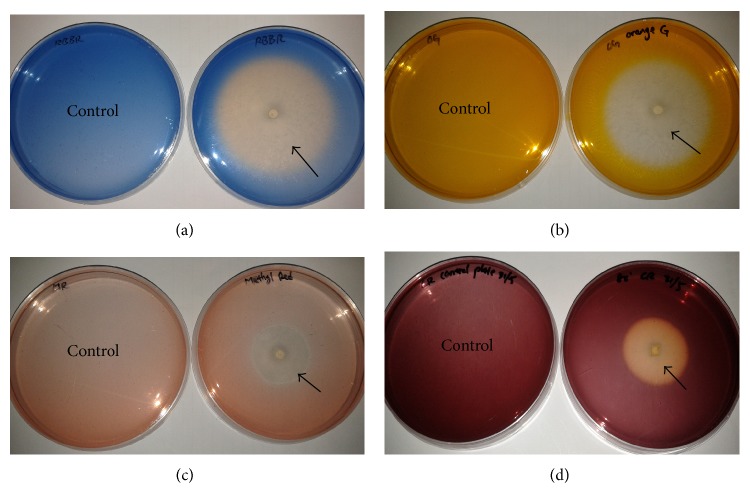
Dye decolourisation by isolate MS8 resulting in the formation of halo (arrow) on agar plate medium containing (a) RBBR, (b) Orange G, (c) Methyl red, and (d) Congo red after four days.

Only one endophyte, isolate MS8, was able to decolourise all four different dyes tested (Figure 1). Dye decolourisation by isolate MS8 was also the most rapid among all of the 20 fungal isolates tested. Both RBBR and Orange G dye in the agar medium started to decolourise within 2 to 3 days after fungal inoculation and were completely decolourised within a period of 8 days with no visible traces of dye adsorption on the fungal mycelium. Congo red and Methyl red were also partially decolourised by isolate MS8 with the production of halo, and no observable dye was absorbed by the fungal mycelium. Isolates MS4 and MS17 were also able to completely decolourise RBBR by day 14 and Orange G by day 12, respectively, with no dye adsorption to the fungal mycelium.

**Figure 2:**
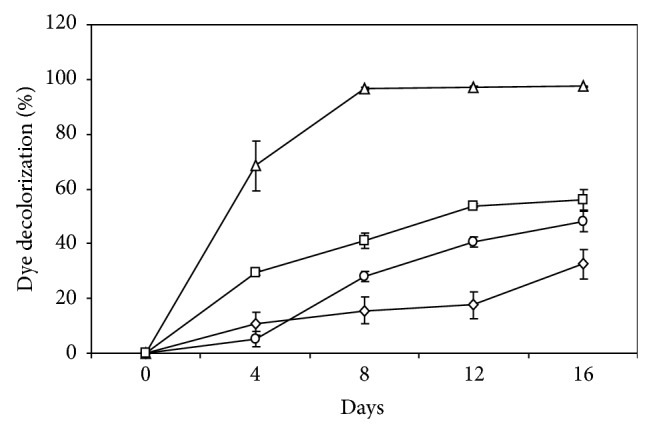
Percentage decolourisation of RBBR (△), Orange G (*◊*), Congo red (⚪), and Methyl red (□) by isolate MS8 in the glucose minimal liquid medium within a period of 16 days.

Isolate MS8 was selected for further dye decolourisation in glucose minimal liquid medium based on the ability to decolourise all the dyes on the agar plate. The results obtained showed that isolate MS8 was able to decolourise all of the four different dyes tested to a different extent (Figure 2). 

Among the four different dyes tested, RBBR was decolorized the fastest and to the greatest extent by isolate MS8, up to 97.4% decolourisation in 4 days. The three azo dyes were also decolourised by isolate MS8 with Methyl red being the highest, reaching 56% decolourisation after 16 days followed by Congo red (48.1%) and Orange G (32.6%). Among the two different dye groups tested, the anthraquinonic dye RBBR was decolourised to a greater extent as compared to the three azo group dyes. Decolourisation of the monoazo dye Methyl red was also greater as compared to the decolourisation of both diazo dyes Congo red and Orange G. This shows that dyes belonging to chemically different groups are not decolourised to the same extent and the structural differences in the dye molecule strongly affect the decolourisation process [17]. Similar results have also been reported on a white rot fungi *Irpex lacteus* which showed a lower efficiency in removal of monoazo and diazo dyes in liquid medium as compared to anthraquinone, phthalocyanine, and triphenylmethane dyes [27]. Decolourisation of a monoazo dye, Xylidine Ponceau, by a basidiomycetous fungal isolate RCK-3 was also reported to be more efficient than the diazo dye, Congo red [28]. As decolourisation of a dye requires the destruction of the chromophore, the complexity and amount of chromophore a dye carries in its chemical structure therefore determine the resistance of the dye towards decolourisation [29]. Decolourisation of the monoazo dye Methyl red was also greater as compared to the decolourisation of the diazo dye Congo red and monoazo dye Orange G even though all three are of the same azo type dye. 

Decolourisation of Orange G by isolate MS8 in liquid medium however was surprisingly lower than the extent of Orange G decolourisation achieved by the isolate on agar plate. A possible reason for this would be that the presence of dye in liquid medium was much more toxic towards the isolate as compared to the toxicity of dye on agar medium. Further quantification of the biomass produced by isolate MS8 with the presence of the tested dye in liquid medium shows that the biomass produced was greatly reduced as compared to the control biomass of the isolate produced in the absence of the dye (Table 2).

The presence of azo type dye in liquid medium has previously been reported to reduce gas solubility, and therefore high azo dye concentration can decrease fungal growth [30]. A white rot fungus *Ischnoderma resinosum*, capable of degrading a wide spectrum of chemically and structurally different synthetic dyes, has also been reported to be more sensitive to dyes in liquid culture than in agar plate [17]. The biomass produced by *I. resinosum* in media containing Malachite Green and Crystal Violet also reached only about 10% when compared to the control biomass produced in the media without dyes [17]. Besides that, the production of some other dye oxidizing activities by the isolate might also be better on agar as compared to liquid medium [31]. 

### 3.3. Fungal Characterisation and Identification

**Figure 3:**
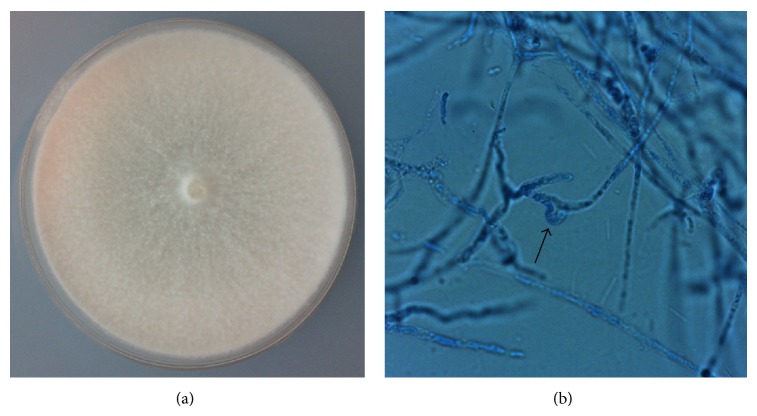
Endophytic fungal isolate MS8. (a) Fungal colony cultured on malt extract agar for 7 days. (b) Microscopic view of the isolate showing fungal hyphae with the production of a clamp connection (arrow).

Isolate MS8 was selected for further identification based on its morphological characteristic and also by comparison of the ITS sequences. Isolate MS8 is a fast growing fungus with mycelium that covers the whole 90 mm Petri dish in 7 days (Figure 3(a)). Aerial mycelium was constantly white, flat, and smooth with no pigment production. The fungal isolates, when observed microscopically, showed the presence of clamp connection (Figure 3(b)), the characteristic feature of basidiomycetous fungi [28]. The isolate however did not produce any spore like structures, which normally provided the basis for fungal identification. This therefore prevents further identification based on the fungal morphological characteristic. Isolation of a mycelial sterilia, fungi that do not sporulate in culture, such as isolate MS8 however is not uncommon during the isolation of endophytic fungi. For a given host, mycelial sterilia can take up to an average of 20% of the population of fungal endophytes [32].

**Figure 4:**
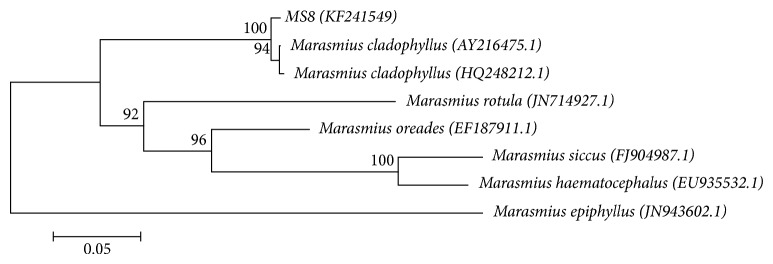
Neighbour-joining tree from ITS sequences showing the relationship between isolate MS8 and other closely related *Marasmius* species retrieved from the GenBank (accession number). Bootstrap values >70% (1000 replicates) are shown on the branches. Bar = 5 nucleotide substitutions per 100 nucleotides.

PCR amplification of the ITS region of isolate MS8 using a universal primer pair, ITS1, and ITS4, resulted in a PCR product with an approximate size of 620 bp in molecular weight. Comparison of the ITS sequence with the established fungal ITS sequences in the GenBank through a standard nucleotide-nucleotide BLAST homology search shows that isolate MS8 shared 99% sequence similarity with a Basidiomycetes, *Marasmius cladophyllus*. The sequence had been successfully deposited in Genbank database (Accession number: KF241549). A phylogenetic tree was constructed to determine the phylogenetic relationship of isolate MS8 with other *Marasmius* species using *M. epiphyllus* as the outgroup (Figure 4). The short branches and clustering of isolate MS8 together with *M. cladophyllus* show that isolate MS8 was closely related to *M. cladophyllus*. This is also supported by a very high bootstrap value of 100. Isolate MS8 was also distantly related with *M. rotula*. 

On a whole, endophytic fungus MS8 identified to be *M. cladophyllus* was able to decolourise the 4 different dyes tested especially anthraquinonic dye which was known to resist degradation due to their fused aromatic structures [33]. The dye decolourization ability of this isolate was also comparable to that of white rot fungi such as *I. lacteus* and *Thelephora* sp [27, 34]. Not much study was reported on the use of *Marasmius* sp. particularly *M. cladophyllus* for dye decolourisation besides screening for ligninolytic activity using RBBR dye [35]. Novel peroxidases capable of degrading beta-carotene were recently found to be produced by* Marasmius scorodonius *[36]. These novel peroxidases may be assigned to the group of “DyP-type” peroxidases (dye decolourising peroxidases) that is able to degrade synthetic anthraquinone dyes [37]. These novel peroxidases may also be produced by isolate MS8 which was shown to effectively decolourise the anthraquinonic dye, RBBR. Further enzymatic studies therefore are necessary in order to elucidate the possible enzymatic activities involved in dye decolourisation by our *M. cladophyllus* isolate MS8. 

Endophytic fungi isolated from the medicinal plant *M. malabathricum* in particular isolate MS8 was able to decolourise both the anthraquinone and azo type of synthetic dye to a different extent. This therefore suggests, that besides white rot fungi, endophytic fungi were also capable of decolourising the synesthetic dyes and of interesting potential to be used in the decolourisation of dyestuff effluents.

## Figures and Tables

**Table 1:** Decolourisation of RBBR, Orange G, Congo red, and Methyl red on agar plate by the twenty locally isolated endophytic fungi after 16 days of cultivation.

Fungal Isolates	Dye decolourisation			
	RBBR	Orange G	Congo Red	Methyl Red
MS1	−	−	−	−
MS2	−	−	−	−
MS3	−	−	−	−
MS4	++	−	+	+
MS5	+	−	−	−
MS6	+	−	+	+
MS7	−	−	−	−
MS8	++	++	+	+
MS9	−	−	−	−
MS10	+	−	+	+
MS11	+	−	+	+
MS12	−	−	−	−
MS13	+	−	+	+
MS14	+	−	+	+
MS15	+	−	+	−
MS16	+	−	+	−
MS17	−	++	+	−
MS18	+	−	+	+
MS19	+	−	+	−
MS20	+	−	+	+

(−): no dye decolourisation; (+): partial or weak dye decolourisation; (++): complete dye decolourisation.

**Table 2:** Fungal biomass produced by isolate MS8 in glucose minimal liquid medium containing RBBR, Orange G, Congo red, and Methyl red within a period of 16 days. Liquid medium with no dye added was used as a control for fungal biomass.

Dyes	Fungal biomass produced (mg mL^−1^)				
	Day 0	Day 4	Day 8	Day 12	Day 16
RBBR	**0.07** ± 0.03	**0.22** ± 0.03	**0.95** ± 0.13	**0.30** ± 0.10	**0.33** ± 0.03
Orange G	**0.07** ± 0.03	**0.12** ± 0.08	**0.23** ± 0.06	**0.37** ± 0.03	**0.30** ± 0.15
Congo Red	**0.08** ± 0.03	**0.15** ± 0.05	**0.22** ± 0.06	**0.38** ± 0.03	**0.55** ± 0.10
Methyl Red	**0.07** ± 0.03	**0.10** ± 0.00	**0.18** ± 0.08	**0.20** ± 0.05	**0.38** ± 0.13
Control (no dye)	**0.07** ± 0.03	**0.43** ± 0.08	**3.72** ± 0.46	**8.17** ± 0.38	**5.47** ± 0.93

Values are mean ± SE (*n* = 3).
